# Co-morbidity of epilepsy in Tanzanian children: A community-based case–control study

**DOI:** 10.1016/j.seizure.2011.10.011

**Published:** 2012-04

**Authors:** Kathryn Burton, Jane Rogathe, Roger G. Whittaker, Kshitij Mankad, Ewan Hunter, Matthew J. Burton, Jim Todd, Brian G.R. Neville, Richard Walker, Charles R.J.C. Newton

**Affiliations:** a Kilimanjaro Christian Medical Centre, Moshi, Tanzania; b Neurosciences Unit, Institute of Child Health, University College London, United Kingdom; c Department of Clinical Neurophysiology, Royal Victoria Infirmary, Newcastle upon Tyne, United Kingdom; d Northumbria Healthcare NHS Foundation Trust, North Tyneside General Hospital, North Shields, United Kingdom; e Great Ormond Street Hospital, London, United Kingdom; f Department of Clinical Research, Faculty of Infectious and Tropical Diseases, London School of Tropical Medicine, United Kingdom; g National Institute for Medical Research, PO Box 1462, Mwanza, Tanzania; h Department of Paediatrics, Muhimbili University of Health and Allied Sciences, Dar-es-Salaam, Tanzania; i Department of Psychiatry, University of Oxford, United Kingdom

**Keywords:** Epilepsy, Africa, Children, Co-morbidity, Cognitive impairment, Education

## Abstract

**Purpose:**

To define the prevalence and associations of co-morbidity and school attendance in older children with epilepsy (CWE) from a rural district of Tanzania by conducting a community-based case–control study.

**Methods:**

Children aged 6–14 years old with active epilepsy (at least two unprovoked seizures in the last five years) were identified in a cross-sectional survey in Tanzania. Co-morbidities were assessed and cases were compared with age-matched controls.

**Results:**

Co-morbidity was very common amongst cases (95/112, 85%), with 62/112 (55%) having multiple co-morbidities. Co-morbidities consisted of cognitive impairment (72/112, 64%), behaviour disorder 68/112 (61%), motor difficulties 29/112 (26%), burns and other previous injuries (29/112, 26%). These complications were significantly more common in cases than in controls (odds ratio 14.8, 95%CI 7.6–28.6, *p* < 0.001). Co-morbidity in CWE was associated with structural cause, abnormal electroencephalogram and early onset seizures. Cognitive impairment was very common in CWE (64%) and was not associated with Phenobarbital use but was associated with motor difficulties, early onset and recurrent seizures. Poor school attendance was found in 56/112 (50%) of CWE, but not in the controls: it was associated with the presence of multiple co-morbidities, especially with motor difficulties in CWE.

**Conclusion:**

Children with epilepsy in a rural area of sub-Saharan Africa had a high level of co-morbidity. Cognitive impairment and poor school attendance were very common. These associated difficulties in CWE in the region need to be addressed to reduce the negative impact of epilepsy on these children.

## 1 Introduction

The incidence of epilepsy is much higher in low and middle income countries (LMICs) compared to high income countries (HICs).^1^ In LMICs, the incidence of epilepsy is 100–190/100,000 per year^2^ with the highest rates in children up to 18 years of age.^3^ It is increasingly recognised that co-morbidities with epilepsy may form a large part of the burden of epilepsy. Co-morbidities (the greater than coincidental association of two conditions in the same individual) in children with epilepsy (CWE) are common in HICs. They include disabling chronic conditions such as cognitive impairment, neuropsychiatric conditions and motor problems.^4^ The impact of these co-morbidities can be lifelong and significantly affect psychosocial outcome^5^ and reduce quality of life for CWE.^6^

There are only a few, mostly observational, studies on co-morbidities in CWE from LMIC.^7–12^ There have been no previous community-based studies in sub-Saharan Africa (SSA) reporting on factors associated with the common co-morbidities found in CWE. Therefore we investigated the prevalence, type and associations of co-morbidities in CWE identified during a community based survey in a rural part of Tanzania.

## 2 Materials and methods

### 2.1 Study area and population

We conducted a cross-sectional study of epilepsy in Hai district, Northern Tanzania by identifying all 6–14 year old children with epilepsy through a door-to-door survey. We used age-matched controls selected from Hai for comparison as previously described (Burton et al., in press).

### 2.2 Definitions

We used the International League Against Epilepsy (ILAE) definitions and defined active epilepsy as two or more afebrile seizures, 24 h apart, unrelated to acute infection, metabolic disturbance, neurological disorders or drugs, in the last five years.^13^ Children who were on antiepileptic drugs were also considered to have active epilepsy. Epileptic seizures were classified according to the ILAE guidelines.^14^ The aetiology of seizures was categorised as idiopathic or structural if there was sufficient evidence from history and examination to assess for an underlying cause for epilepsy and as undetermined if there was insufficient data.^15^

### 2.3 Subject ascertainment and criteria for inclusion and exclusion

During a census in January 2009 a previously validated questionnaire to identify epilepsy was administered to all households in Hai district^16^ and village enumerators were also trained to identify likely cases of epilepsy. The study paediatrician (KJB), who has training in paediatric epilepsy, assessed each child. The diagnosis of active epilepsy and seizure type were verified by paediatric neurologists (CN and BN). For this study, cases of epilepsy were defined as children with active epilepsy aged 6–14 years who were resident in Hai at the census. Those children for whom consent was refused or who were below 6 years old (to eliminate any children with febrile seizures) were excluded. Controls were drawn from a random computer generated sample selected from all the children aged 6–14 years who were resident in Hai at the census in 2009. Controls were identified through the census by matching for age (±1 year), sex and village to the positive responders. From this list of eligible children, we estimated that 186 controls were required to account for the likely 25% refusal rate, to give at least one control for each case.

### 2.4 Neuropaediatric assessment

A full clinical history was taken using a standardised questionnaire and a neuropaediatric examination was completed for each case and control. All probable epilepsy cases and controls were recalled for further assessment. For cases, clinical history and response to treatment were reviewed.

An electroencephalogram (EEG) and computerised tomography (CT) scan were offered to every case at recall. EEG was performed using a Nihon Kohden (Japan) Neurofax 11000K machine. 20 leads were attached using a standard montage. Patients had EEGs whilst awake with hyperventilation and photic stimulation. Most patients also had recordings whilst asleep, A UK neurophysiologist (RW) reported EEGs using a standardised form. The CT scans were performed with contrast on a Philips Tomoscan 4000 machine. CT scans were reported locally to exclude acute pathology and were then reported using a standardised format by a paediatric neuroradiologist (KM) in the UK.

An assessment of cognitive function was made using the Goodenough–Harris Drawing Test.^17^ Those scoring less than 70 (>2 standard deviations below the mean) were categorised as having cognitive impairment. The Goodenough–Harris Drawing Test was used in this study to compare cognitive function as it has been shown to have good reliability and validity compared to other tests of intelligence.^18,19^

The Rutter questionnaire was used for assessing behaviour.^20^ Many of the cases did not attend school so the parent scale was used to give a comparable score for all children. Children with total scores of 13 or more were designated as showing behaviour disorder.

History of injuries, motor and feeding difficulties were assessed from the history and examination. A child was designated as having feeding difficulties if they presented with at least one of the following: coughing, choking or taking more than half an hour at meal times. Any child who was not attending school regularly or not at all was designated to have poor school attendance. We measured Snellen visual acuity at six metres using letter matching in each eye separately. A child was designated as having visual impairment if visual acuity was less than 6/12 in either eye. We assessed auditory discrimination from three metres behind the subject.

## 3 Ethical approval

Approval for this study was obtained from the National Institute for Medical Research in Tanzania and locally from the Ethics Committee of Kilimanjaro Christian Medical College, Moshi. Parents and guardians were given written and verbal information in Kiswahili before signing consent forms agreeing to participation. Children with conditions requiring treatment or referral were referred appropriately to local services.

### 3.1 Data analysis

All data were double entered into a Microsoft Access (2007, Microsoft Corporation, Redmond, WA) database. The two database copies were compared using Epidata (Version 3.1, Epidata Association, Denmark) and each discrepancy was checked against original data forms. Statistical analysis was performed using STATA v.10 (Statacorp, College Station, TX, USA).

Univariate odds ratios (OR) with 95% confidence interval (95%CI) were calculated for associations between epilepsy and co-morbidity (comparing none to one or multiple co-morbidities with burns and injuries excluded) using ordinal logistic regression. Ethnic groups were classified as Chagga (the predominant group) against the others. Education of head of household was a surrogate marker for socio-economic status as previous research showed that this was a key determinant in explaining the between-household variation in expenditure.^21^ Early-onset epilepsy was defined as that which started at or before the age of three years. Cases were labelled as having recurrent seizures if they had any ongoing seizures in the three months before follow-up. Multivariable logistic regression models were developed for cases including factors with *p*-values less than or equal to 0.2 in the univariate analysis.

## 4 Results

### 4.1 Study subjects

Overall 112 children with active epilepsy and 113 controls were identified (Supplementary Fig. 1). For one case only (girl, aged 10 years) carers refused consent. Demographic characteristics are presented in Table 1; age, sex and head of the household education were comparable with no significant difference between cases and controls. The proportion of cases from the Chagga ethnic group was lower in cases than for controls (OR 2.5, 95%CI 1.2–5.3, *p* = 0.014), mainly because of greater refusal in consent. However, those CWE who were Chagga were not significantly different to others in terms of sex, age, education of household head or on seizure variables. Cases were less likely to have both parents residing at home. Of 186 computer selected potential matched controls, 73 (39%) were either not found or their carer refused consent. There was no significant difference between controls who were seen and controls who were not in terms of age and sex (median age 11 years for both, *t*-test *p* = 0.39; and 52.2% and 46.6% were male, respectively, OR 1.3, 95%CI 0.7–2.3, *p* = 0.45; other variables were not available for analysis).

In cases, the probable aetiology from the clinical history was idiopathic in 56/112 (50.0%), hypoxic ischaemic encephalopathy in 10/112 (8.9%), intracranial infection in 9/112 (8.0%), head injury 3/112 (2.7%), neurocutaneous disorder (one each of tuberous sclerosis, neurofibromatosis and Sturge–Weber) in 3/112 (2.7%), other disorders in 19/112 (17.0%) and undetermined in 12/112 (10.7%). 13/112 (11.6%) had a positive family history of non-febrile seizures. An episode of status was reported in 32/112 (28.6%), had not occurred in 70 (62.5%) and was not known in 10 (8.9%). Onset of seizures was before 6 years of age in 71/112 (63.4%).

### 4.2 Prevalence and types of co-morbidity

**Fig. 1:**
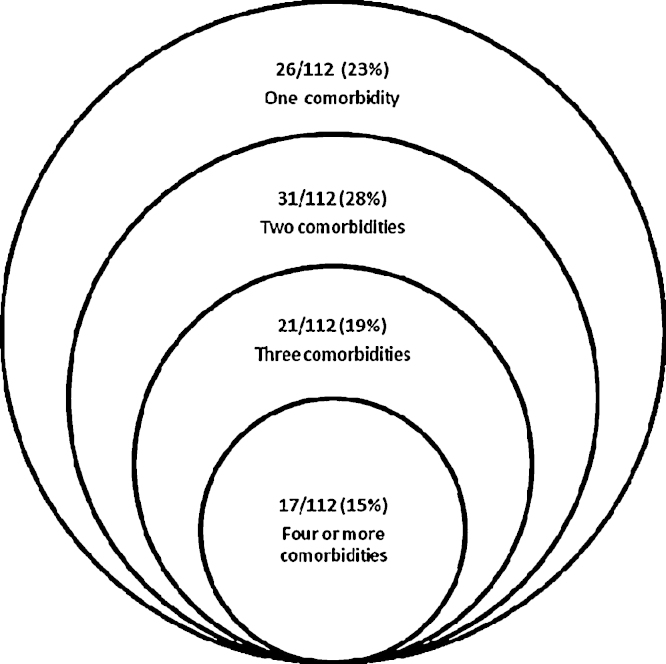
Overlap of co-morbidities in children with epilepsy.

The prevalence of co-morbidity was high in CWE; 95 (84.8%) had one or more co-morbidities compared to 31 (27.4%) controls (mostly behavioural problems); 69 (61.6%) CWE had multiple comorbid conditions compared to 4 (3.5%) of controls. Any or multiple co-morbidities were significantly more common in the cases than in the controls; OR 14.8, 95%CI 7.6–28.6, *p* < 0.001. The range of co-morbidities in cases and controls are shown in Table 2 and Fig. 1.

Motor difficulties were present in 29 (25.9%) CWE and of these, 19 (65.5%) had cerebral palsy. In those with motor difficulties, all had multiple comorbid conditions and 14 (48.2%) had four or more co-morbidities. In 10 (8.9%) cases there were feeding difficulties of whom 9 had associated cerebral palsy and 1 had an unclassified seizure disorder with regression. Disordered behaviour was found in 66% of cases compared to 19% controls, the latter mostly had mildly disordered behaviour (Burton et al., in press).

### 4.3 Associations with co-morbidity

The factors associated with co-morbidity in cases on univariate analysis are shown in Table 3. On multivariable regression modelling, the independent factors associated with co-morbidity in CWE were structural aetiology, abnormal EEG and early onset of seizures.

### 4.4 Prevalence and associations of cognitive impairment in cases

Cognitive impairment was very common in CWE and was markedly more common in cases than in controls (72 (64.3%) in cases vs 6 (5.3%) in controls; OR 37.2, 95%CI 14.7–94.2, *p* < 0.001). Cognitive impairment was not associated with treatment type (Table 4). The independent factors associated with cognitive impairment in CWE in the multivariable model were motor difficulties, early onset and recurrent seizures.

### 4.5 Prevalence and associations of school attendance

All the controls attended school regularly. However, 56/112 (50%) CWE were not attending school regularly. Of these, 52/56 (93%) had one or more co-morbidities compared to 41/54 (76%) CWE who did attend school regularly (OR 4.1, 95%CI 1.3–13.6, *p* = 0.020). CWE with co-morbidities were much less likely to attend school (single OR 4.7, 95%CI 1.1–19.1, *p* = 0.033; multiple OR 50.2, 95%CI 14.2–177, *p* < 0.001). Associations with poor school attendance are shown in Table 5. As structural cause, early onset and recurrent seizures were strongly associated with presence of co-morbidity, they were excluded from the multivariable analysis. In multivariable analysis the presence of multiple co-morbidities, especially motor difficulties, and poor education of the household head remained significantly associated with poor school attendance.

## 5 Discussion

Co-morbidities in CWE are increasingly recognised as an important issue as they may adversely affect children even more than the seizures themselves in terms of poor social long-term outcome^22^ and reduced quality of life.^6^

### 5.1 Prevalence and types of co-morbidities in HICs and LMICs

There have only been a few community-based studies of the prevalence and type of co-morbidities in CWE worldwide. The largest study was a Finnish study of CWE^5^; behaviour problems were found in 58%, communication difficulties in 78%, mobility problems in 73% and learning difficulties were found in 76%. Another Finnish population-based study of CWE found additional neurological co-morbidity in 40% and learning difficulties in 31%.^23^ A population-based study from Canada reported that 21% of CWE had mental retardation.^24^ The prevalence of co-morbidity in our community-based study matched that from the largest study from Finland but compared to the smaller studies, we found more cognitive impairment amongst CWE and included other co-morbidities such as behaviour disorder.

Few previous population studies from LMICs have studied the prevalence and types of co-morbidity in CWE. A cross-sectional survey from Kenya found 34/110 (31%) children with lifetime epilepsy had moderate to severe neurological impairments, 22 (20%) had cognitive impairment and 5 (5%) motor impairment.^11^ In a separate study of Kenyan adults and children with active convulsive epilepsy, 27% had cognitive impairment.^12^ In our study, the prevalence of any co-morbidity was higher; this is because in our study, behaviour disorder and feeding difficulties were also included and cognitive and motor difficulties were more common. Reported rates of co-morbidities in CWE in studies from SSA may be a relative underestimate compared to HICs. Children in SSA with severe neurological impairments tend not survive, as shown in Kenya where they found an increased mortality rate in children with severe neurological deficits after severe malaria.^25^ Differences between studies may also be due to variations in populations, definitions and assessment tools.

### 5.2 Associations of co-morbidity in CWE

Comorbid conditions are likely to be caused by underlying brain disorder. The evidence for this is from studies that show that cognitive impairment,^26^ Attention Deficit Hyperactivity Disorder^27^ and other behavioural problems^28^ predate the onset of seizures in children. Our study design was not able to distinguish if co-morbidities pre- or postdated the onset of epilepsy. Symptomatic aetiology, abnormal EEG and early-onset seizures were associated with co-morbidities in our study. They are all more likely to be seen with underlying brain disorder and thus with co-morbidities.

### 5.3 Prevalence of cognitive impairment in HICs and LMICs

In HICs the reported prevalence of cognitive impairment in CWE ranges from 31 to 76%.^5,29–31^ Our study found that cognitive impairment was common (64%) matching the higher recorded prevalences in HICs.

There are only two previous studies reporting on cognitive function in CWE in sub-Saharan Africa, both from Kenya. A study of children with active convulsive epilepsy, found that moderate to severe cognitive impairment (assessed clinically) was present in 27%.^12^ The other cross-sectional study of children with lifetime epilepsy, found 20% had cognitive impairment.^11^ In these studies, children with mild to moderate cognitive impairment may not have been identified and in the second, were not included. This together with different assessment tools and population characteristics may account for the lower reported rate of cognitive impairment compared to our study.

### 5.4 Associations of cognitive impairment in CWE

Multiple factors may account for the strong association between cognitive impairment and epilepsy in our study. Cognitive impairment may be part of the underlying brain disorder, in some cases cognitive impairment may be made worse by seizure activity or it could be related to antiepileptic drug use.

There is good evidence that cognitive impairment is part of the underlying brain disorder in CWE. It has been shown that cognitive impairment and cerebral palsy, which are both seen with brain disorders, are associated with an increased risk of developing epilepsy.^32,33^ In new-onset epilepsy, cognitive impairment has been shown to pre-date seizure onset.^34^ Cognitive impairment can also be made worse by abnormal electrical brain activity.^35^ Patients in whom seizures are refractory^36^ and with some syndromes, can have cognitive decline.^37^

Most children were treated in our study, but those that were on AED, were taking Phenobarbital (PB), which could be postulated as the cause for the high prevalence of cognitive impairment. Early studies found a worrying association between Phenobarbital use for febrile seizure prophylaxis and cognitive problems but more recent masked trials have shown no adverse effect on cognition in children with epilepsy.^38^ There was no significant association between type of treatment and cognitive impairment in our study and PB did not seem to have a significant detrimental effect on cognition in these CWE.

The association in our study between cognitive impairment and epilepsy is likely to be multifactorial. Cognitive impairment was associated with motor difficulties, early onset and recurrent seizures. These are more likely to be found with underlying brain disorder and therefore with cognitive impairment which concurs with previous studies. Further studies on the nature and associations of cognitive impairment in CWE in SSA would be helpful.

### 5.5 Impact of co-morbidity

Co-morbidities have been found to have a major impact on CWE in terms of quality of life. Studies have found that neurological co-morbidities had an adverse impact on health-related quality of life.^6,39^ Co-morbidities have also been shown to have an adverse effect on the lifelong social outcomes for CWE even if seizures remit.^22^ Studies from across the world have shown that those with childhood epilepsy received less education and were less likely to be employed or married especially if they had associated cognitive impairment.^40–42^ These studies concur with our study that found that school attendance was poor in CWE, but not in controls and was associated with the presence of co-morbidity particularly with motor difficulties and to some extent with behaviour difficulties. The effect on access to school of less education of the household head may be causal in terms of income or of value placed on accessing education. Access to specialist education for children with cognitive impairment is very limited in Tanzania as teachers are not able to meet special needs in mainstream schools, there are only a few special schools and most of these are in urban areas.

### 5.6 Sources of bias

To minimise ascertainment bias in this community-based study we aimed to identify all cases of epilepsy in children aged 6–14 years in the study area by using a validated screening questionnaire and trained enumerators. Case status was defined by two paediatric neurologists. If children were unavailable initially, repeat visits and transport to assessment were offered. Controls were well matched in terms of age, sex and socioeconomic status and came from the same population. Cases were less likely to have both parents at home but this was probably because parents tended to leave children with disabilities with relatives. More cases were from non-Chagga groups compared to controls but did not differ on the main variables from cases from the Chagga group. Genetic or lifestyle differences may have had a small effect on the presence of co-morbidities but similar ethnic groups were represented in both cases and controls. Although the control group was randomly chosen, it may not have been entirely representative of the general population as there were more refusals amongst the potential controls in the non-Chagga ethnic groups. However follow up rate was high and drop-out rate was similar in both cases (7.1%) and controls (11.5%). Both cases and controls were assessed using identical methods and questionnaires. The assessors could not be blinded to case status but were unaware of seizure variables and assessment scores.

## 6 Conclusion

Children with epilepsy in a rural area of sub-Saharan Africa had a very high level of co-morbidity, many with multiple co-morbidities, particularly cognitive impairment and poor school attendance. These associated difficulties in CWE in the region need to be addressed to reduce the negative impact of epilepsy on the life chances of these children.

## Conflict of interest

None of the authors has any conflict of interest to disclose.

## Figures and Tables

**Table 1:** Characteristics of children with epilepsy and controls.

Characteristics	Cases (*N* = 112)		Controls (*N* = 113)		Chi squared
	*N*	(%)	*N*	(%)	*p*-Value
Sex					
Male	57	(50.9)	57	(50.4)	*p* = 0.946
Female	55	(49.1)	56	(49.6)	
Age at assessment (years)					
Less than 12 years	73	(65.2)	75	(66.4)	*p* = 0.850
12 years and over	39	(34.8)	38	(33.6)	
Ethnic group (Chagga)					
Chagga	86	(76.8)	101	(89.4)	*p* = 0.012
Other	26	(23.2)	12	(10.6)	
Religion (Christian)					
Christian	91	(81.3)	90	(79.7)	*p* = 0.762
Muslim and other	21	(18.7)	23	(20.3)	
Parents resident at home					
Both	71	(63.4)	89	(78.8)	*p* = 0.025
One parent	26	(23.2)	18	(15.9)	
None	14	(12.5)	5	(4.4)	
Not known	1	(0.9)	1	(0.9)	
Education of head of house					
None	6	(5.4)	3	(2.7)	*p* = 0.196
Primary	93	(83.0)	90	(79.6)	
Secondary	11	(9.8)	12	(10.6)	
Not known	2	(1.8)	8	(7.1)	

**Table 2:** Co-morbidities in cases and controls.

	Cases (*N* = 112)		Controls (*N* = 113)	
	*N*	(%)	*N*	(%)
**Co-morbidity**				
None	17	(15.2)	82	(72.6)
One	26	(23.2)	27	(23.9)
Multiple	69	(61.6)	4	(3.5)
**Type of co-morbidity**				
Motor difficulties present	29	(25.9)	0	(0)
No motor difficulties	83	(74.1)	113	(100)
Accidental burns and injuries	18	(16.1)	7	(6.2)
No other accidental injuries	94	(83.9)	106	(93.8)
Burns form seizures	11	(9.8)	0	(0)
Cognitive impairment				
Present	72	(64.3)	6	(5.3)
None	30	(26.8)	93	(82.3)
Not known	10	(8.9)	14	(12.4)
Behavioural problems				
Present	68	(60.7)	19	(16.8)
None	36	(32.1)	80	(70.8)
Not known	8	(7.2)	14	(12.4)
Hearing impairment				
Present	0	(0.0)	1	(0.9)
None	100	(99.1)	112	(99.1)
Not known	1	(0.9)	0	(0)
Visual impairment				
Present	11	(9.8)	2	(1.8)
None	100	(89.3)	111	(98.2)
Not known	1	(0.9)	0	(0)
Feeding problems				
Present	10	(8.9)	0	(0)
None	101	(90.2)	113	(100)
Not known	1	(0.9)	0	(0)

**Table 3:** Factors associated with co-morbidity (not including burns and injuries) in cases.

Variable	OR	95%CI	*p*-Value
**Univariate associations**			
Sex (male)	1.0	0.5–2.0	0.935
Age at assessment (years)	0.4	0.2–0.9	0.027
Ethnic group (not Chagga)	0.9	0.4–2.1	0.798
Parents resident at home			
Both	1.0	–	–
One	1.6	0.7–3.0	0.311
None	1.5	0.5–4.4	0.498
Education of head of house			
None	1.0	–	–
Primary	0.3	0.03–2.6	0.265
Secondary	0.2	0.02–2.3	0.208
History of status (present)	0.7	0.3–1.6	0.385
Age at onset (3 years or less)	3.4	1.6–7.4	0.002
Recurrent seizures (present)	3.8	1.7–8.6	0.001
More than one seizure type (present)	2.0	0.6–6.9	0.255
Causal (structural)	5.3	2.2–12	<0.001
Abnormal EEG	4.7	2.0–11	<0.001
Abnormal CT scan	2.9	1.0–8.1	0.044
**Multivariable logistic regression model**			
Age at assessment (years)	0.9	0.3–2.4	0.777
Age at onset (3 years or less)	3.1	1.2–8.5	0.024
Recurrent seizures (present)	2.5	0.9–7.0	0.094
Causal (structural)	4.5	1.4–12.6	0.013
Abnormal EEG	4.1	1.4–12.5	0.012
Abnormal CT scan	2.0	0.6–6.6	0.277

**Table 4:** Factors associated with cognitive impairment in CWE.

Variable	OR	95%CI	*p*-Value
**Univariate associations**			
Sex (male)	0.8	0.3–1.8	0.574
Age at assessment (years)	0.8	0.3–1.8	0.521
Ethnic group (not Chagga)	1.0	0.4–2.8	0.976
Parents resident at home			
Both	1.0	–	–
One	1.7	0.6–4.8	0.346
None	2.6	0.5–13	0.239
Education of head of house (none/primary only)	2.6	0.7–9.9	0.150
Age at onset (3 years or less)	4.8	1.9–12	0.001
History of status (present)	1.6	0.6–4.5	0.381
Recurrent seizures (present)	2.5	1.0–6.1	0.044
More than one seizure type (present)	2.5	0.5–12	0.248
Structural cause	2.3	0.9–5.9	0.076
Motor difficulties	17.4	2.2–135	0.006
Focal abnormalities on EEG and/or CT scan	3.6	1.4–9.4	0.010
Treatment type			
None	1.0	–	–
Carbamazepine or Valproate	0.2	0.0–1.9	0.154
Phenobarbital	2.0	0.8–5.2	0.147
Polytherapy	3.8	0.4–33	0.226
**Multivariable logistic regression model**			
Education of head of house (none/primary only)	10.8	0.8–143.9	0.071
Age at onset (3 years or less)	6.4	1.9–21.4	0.003
Recurrent seizures (present)	4.2	1.2–14.7	0.023
Structural cause	0.8	0.2–3.2	0.707
Motor difficulties	20.3	1.2–331.7	0.035
Focal abnormalities on EEG and/or CT scan	1.4	0.4–4.8	0.623
Treatment type			
None	1.0	–	–
Carbamazepine or Valproate	0.2	0.0–4.5	0.343
Phenobarbital	2.1	0.6–7.0	0.218
Polytherapy	2.7	0.2–31.0	0.434

**Table 5:** Factors associated with poor school attendance in cases.

Variable	OR	95%CI	*p*-Value
**Univariate associations**			
Sex (male)	1.8	0.8–3.8	0.130
Age at assessment (years)	1.1	0.5–2.4	0.793
Ethnic group (not Chagga)	1.1	0.4–2.6	0.901
Parents resident at home			
Both	1.0	–	–
One	1.2	0.5–3.0	0.677
None	2.0	0.6–6.6	0.247
Education of head of house (none/primary only)	3.1	0.8–12.3	0.111
Age at onset (3 years or less)	2.3	1.1–5.1	0.033
Co-morbidity			
None	1.0	–	–
One	1.1	0.3–3.7	0.978
Multiple	6.7	2.1–21.3	0.001
Recurrent seizures (present)	4.4	1.9–10.0	0.001
Structural	2.4	1.1–5.4	0.029
Motor difficulties	4.0	1.5–10.5	0.004
Behaviour disorder	1.0	1.0–1.1	0.110
Hearing or visual difficulties	1.8	0.5–6.6	0.362
Cognitive impairment	26.4	5.8–120.7	<0.001
**Multivariable logistic regression model**			
Sex (male)	2.1	0.9–5.1	0.104
Education of head of house (none/primary only)	3.0	0.7–14.1	0.158
Co-morbidity			
None	1.0	–	–
One	1.5	0.4–6.0	0.595
Multiple	10.2	2.8–37	<0.001
**Multivariable logistic regression model using individual co-morbidity types**^a^			
Sex (male)	1.5	0.6–3.4	0.393
Education of head of house (none/primary only)	7.2	1.4–38	0.020
Motor difficulties	7.1	2.1–23.5	0.001
Behaviour disorder	1.0	1.0–1.03	0.095
Hearing or visual difficulties	1.8	0.4–7.2	0.425

a In this analysis, binary variables for each of motor difficulty, behaviour disorder and hearing/visual difficulty were inserted in the model to see their individual associations with poor school attendance.

## Appendix A Supplementary data

Supplementary Fig. 1 Flow chart of case ascertainment and recruitment.
