# Supplementary figures and images for: Co-morbidity of epilepsy in Tanzanian children: A community-based case–control study
Source: Seizure. 2012 Apr;21(3):169–74. doi: 10.1016/j.seizure.2011.10.011 (PMC3672980; doi:10.1016/j.seizure.2011.10.011)

**Supplementary Figure 1: Flow chart of case ascertainment and recruitment**

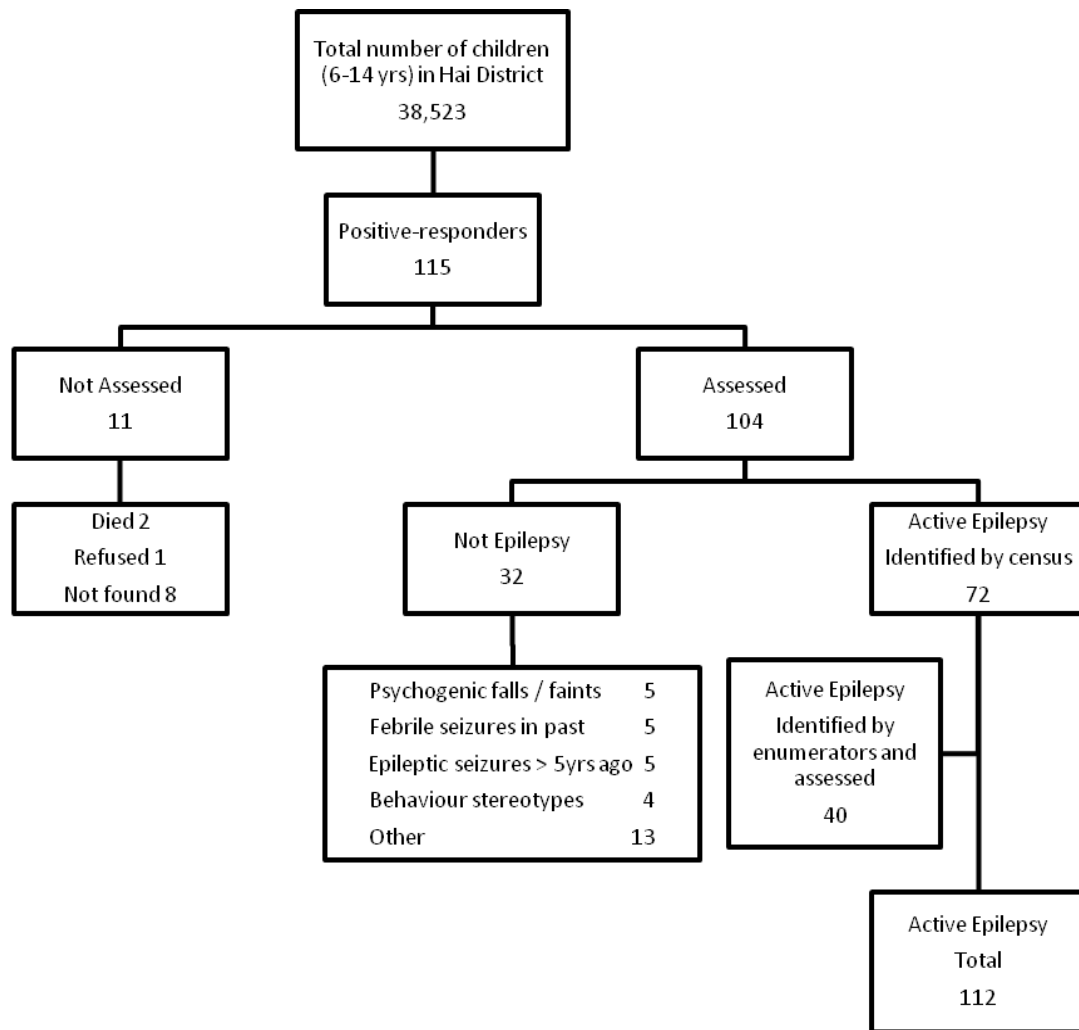

Supplement: Supplementary Fig. 1 — Flow chart of case ascertainment and recruitment. [file mmc1.pdf]
